# Investigation of Precipitation Behavior of a Novel Ni-Fe-Based Superalloy during High-Temperature Aging Treatment

**DOI:** 10.3390/ma17194875

**Published:** 2024-10-04

**Authors:** Jun Cheng, Kejian Li, Zhengang Yang, Xin Huo, Manjie Fan, Songlin Li, Shengzhi Li, Qu Liu, Qingxian Ma, Zhipeng Cai

**Affiliations:** 1 Department of Mechanical Engineering, Tsinghua University, Beijing 100084, China; chengj22@mails.tsinghua.edu.cn (J.C.); kejianli@mail.tsinghua.edu.cn (K.L.); yzghebut@163.com (Z.Y.); maqxdme@mail.tsinghua.edu.cn (Q.M.); czpdme@mail.tsinghua.edu.cn (Z.C.); 2 Shanghai Turbine Plant, Shanghai Electric Power Generation Equipment Co., Ltd., Shanghai 200240, China; huoxin@shanghai-electric.com (X.H.); fanmj@shanghai-electric.com (M.F.); lisl3@shanghai-electric.com (S.L.); lishzh@shanghai-electric.com (S.L.)

**Keywords:** Ni-Fe-based superalloy, advanced ultra-supercritical, high-temperature aging treatment, precipitation behavior, grain boundary migration

## Abstract

The precipitation behavior of a novel Ni-Fe-based superalloy developed for advanced ultra-supercritical (A-USC) coal-fired power plant applications during high-temperature aging treatment was investigated. The results showed that the major precipitates in the novel alloy were randomly distributed MC carbides, M_23_C_6_ carbides at grain boundaries, and the γ′-Ni_3_ (Al, Ti) phase in grain interiors after aging. MC remained relatively stable during both short-term and long-term aging. M_23_C_6_ quickly precipitated and exhibited a discrete distribution at grain boundaries during short-term aging, and partly developed into continuous films during long-term aging. After uniform precipitation, the shape of γ′ remained spherical, and the size kept increasing with aging time according to the Lifshitz–Slyozov–Wagner (LSW) model. The hardness of the novel alloy was mainly associated with the precipitation behavior of γ′; as γ′ gradually precipitated, the hardness steadily increased; after complete precipitation, as the size of γ′ increased, the hardness first increased and then decreased, reaching the peak hardness when the average radius of γ′ achieved the critical size. In addition, the novel alloy exhibited abnormal coarsening behavior at grain boundaries during both short-term and long-term aging. The coarsened grain boundaries were actually precipitate-free zones (PFZs) and the coarsened and elongated rod-like particles inside were identified as γ′ precipitates. The mechanism of strain-induced grain boundary migration and the discontinuous coarsening reaction is proposed for the formation of PFZs. Furthermore, PFZs were considered to be potential crack sources during the creep rupture test, leading to earlier failure of the material.

## 1. Introduction

Developing advanced ultra-supercritical (A-USC) units with high parameters can significantly improve the efficiency of coal-fired power generation and effectively reduce carbon dioxide (CO_2_) emissions [1,2,3]. Conventional ferritic or austenitic heat-resistant steel in current power plants cannot meet the service performance requirements of A-USC units [4,5]. Ni-based superalloys including solution-strengthened, γ′-Ni_3_ (Al, Ti) phase precipitation-strengthened, and γ″-Ni_3_Nb phase precipitation-strengthened types have become the main candidate materials for A-USC units due to their excellent creep strength and corrosion resistance [6,7]. The candidate materials for the high-temperature components of A-USC units that have been developed include Inconel 740/740H [8], Inconel 617/617B [9], Haynes 230/282 [10], and Nimonic 263 [11], among which Inconel 740H and 617B (also known as CCA 617) have the best comprehensive performance. However, 740H and 617B are prohibitively expensive due to the high content of Co, Mo, and W and have poor workability for large components. Recently, the Xi’an Thermal Power Research Institute Co., Ltd. (Xi’an, China) and China Huaneng Group Co., Ltd. (Beijing, China) jointly developed a novel Ni-Fe-based superalloy [12] with high comprehensive cost-effectiveness by increasing the Fe content and reducing the Co content. The novel alloy belongs to Ni-based superalloys strengthened by the γ′ phase, with higher tensile strength than that of the 617B alloy and extremely low Nb content. It can effectively solve the problem of segregation in large thick-walled forgings and is expected to be used for manufacturing high-temperature components of A-USC units.

The γ′ precipitation-strengthened Ni-based superalloys usually contain a large amount of alloying elements, so the precipitates often form a metastable microstructure after heat treatment [13]. Therefore, high-temperature aging for these alloys often results in the evolution of precipitate size, shape, and distribution, even undergoing phase reactions to transform into topologically close-packed (TCP) phases such as σ and η, which significantly affect the mechanical properties of the materials [14]. For instance, with the progression of aging, the size of γ′ gradually increases [14,15,16,17,18,19,20], the shape may undergo a transformation between cubic and spherical [18,19], and TCP phases may be formed in some alloys [14], which in turn degrades the mechanical properties including tensile strength [15,16,18,19,20], toughness [17], and creep strength [14,19]. The evolution of carbides can also have an impact on the strength, plasticity, and fracture behavior of alloys to some extent [21,22,23]. In addition, several previous studies have pointed out that the formation of precipitate-free zones (PFZs) at grain boundaries weakens the creep resistance of alloys [24,25]. Undoubtedly, it can be concluded that ideal properties can be achieved by optimizing the size, shape, and distribution of the precipitates, especially γ′, and controlling harmful phase transformation reactions [26]. Therefore, it is necessary to study the precipitation behavior of Ni-based superalloys during high-temperature aging and further investigate the correlation between the microstructure and mechanical properties to ensure the reliability and safety of high-temperature components in service.

The objective of this paper is to investigate precipitates’ evolution of the novel alloy during high-temperature aging treatment. Precipitation behavior during the precipitation process was mainly studied through the short-term aging treatment of the novel alloy in the solution treatment state, and precipitation behavior during the coarsening process was mainly studied through the long-term aging treatment of the novel alloy in the standard heat treatment state. The precipitation and coarsening of the γ′ phase were focused on and the abnormal coarsening behavior at grain boundaries during aging was studied in detail. In addition, the correlation between precipitation behavior and mechanical properties was elucidated. The research results of this paper can provide reference for subsequent welding research and the development and improvement of novel Ni-based superalloys.

## 2. Materials and Methods

The novel alloy material used in this paper was produced by a Shanghai turbine plant from Shanghai Electric Power Generation Equipment Co., Ltd. using heavy forging technology. After measurement by an electron probe microanalyzer (EPMA, JXA8230, JEOL, Tokyo, Japan), its main chemical composition was determined, as shown in Table 1. To eliminate the inherent dendritic and precipitated phases [12,27], the material underwent solution treatment after forging with parameters of 1080 °C × 3.5 h (air cooling for 0.5 h) + 1000 °C × 2 h (air cooling to room temperature). After two-step aging treatment of the novel alloy in the solution treatment (ST) state at 650 °C × 20 h (cooling rate of 5 °C/h) + 800 °C × 20 h (cooling rate of 5 °C/h), the novel alloy in the standard heat treatment (SHT) state was obtained.

**Figure 1:**
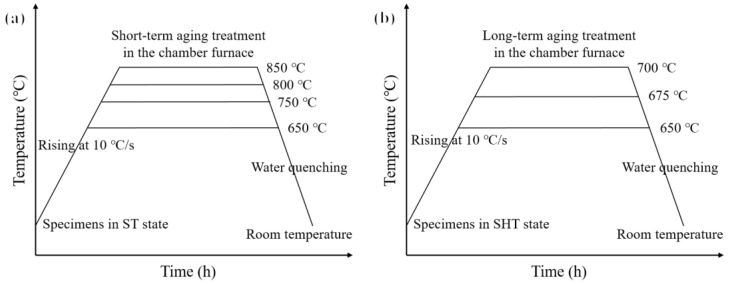
Schematic diagrams of the experimental procedures of (**a**) short-term aging treatment and (**b**) long-term aging treatment.

The aging experiments of the novel alloy were conducted using a chamber furnace. As shown in Figure 1a, considering the temperature range for rapid precipitation of the γ′ phase, the specimens (6 mm × 6 mm × 3 mm) in the ST state were subjected to short-term aging treatment at 650 °C, 750 °C, 800 °C, and 850 °C, respectively. After reaching the preset temperatures at a rate of 10 °C/s, each group of specimens was taken out sequentially and quenched with water to room temperature at an aging time of 1/2/10/60 h based on the real-time precipitation of the γ′ phase. In view of the actual service conditions of the material, as shown in Figure 1b, the long-term aging specimens were in the SHT state before the experiment, and the conditions were 650 °C/5827 h, 675 °C/1615 h, 700 °C/3804 h, and 700 °C/4368 h. In addition, a creep rupture test was performed at 700 °C/230 MPa to study the relationship between abnormally coarsened behavior at grain boundaries and creep rupture failure.

After the aging experiments and creep rupture test, the metallographic specimens were prepared for subsequent microstructure observation and mechanical property testing. After grinding to 3000 grit and polishing to a 2.5 μm surface finish, metallographic specimens were obtained by immersion in an etching solution (composed of 30 mL glycerol + 25 mL hydrochloric acid + 5 mL nitric acid) for about 7 min at room temperature. An optical microscope (OM, OLYMPUS DP72, Tokyo, Japan) was used to observe metallographic specimens and analyze microscopic features at a relatively macroscopic scale (low magnification). A scanning electron microscope (SEM, GeminiSEM 300, ZEISS, Jena, Germany) was used as the main tool for microstructure observation. The acceleration voltage of the SEM was set to 15 kV, the work distance was adjusted to 8.5–9 mm, and the imaging mode was selected as secondary electron mode with a magnification range of 50–50,000×. The average grain size was determined according to the ASTM E112-13 standard [28], and the size of the γ′ phase was calculated by Image J software (version 1.54f). The SEM equipped with energy dispersive spectroscopy (EDS) and EPMA were used to analyze the chemical composition of specific microregions. The crystallographic information of the specimens was characterized by the SEM equipped with an electron backscatter diffraction (EBSD) detector. The specimens for EPMA and EBSD observations were electro-polished using a solution containing 65 mL phosphoric acid + 15 mL sulfuric acid + 12 mL glycerol + 3 mL deionized water + 5 g CrO_3_. After the specimens were prepared by a focused ion beam (FIB), transmission electron microscopy (TEM, Tecnai G2 F20 S-TWIN, SemiStar Corp., Morgan Hill, CA, USA) was used for the morphology observation and EDS and selected area electron diffraction (SAED) were used for analysis.

Microhardness testing was conducted based on the ASTM E92-17 standard [29] by using a microhardness tester (FM-810, FT, Tokyo, Japan) with a 200 gf load held for 10 s. The hardness of each specimen was measured at least seven times to obtain the average value, and the distance between the adjacent measurement points was far enough to another measurement. An in situ nanomechanical testing system (FT-NMT04, Femto Tools, Buchs, Switzerland) installed in the Zeiss SEM was used to test the reduced modulus distribution of a 220 μm × 160 μm region. The continuous stiffness measurement (CSM) method was adopted to measure continuously the reduced modulus as a function of indentation depth with a frequency of 140 Hz and an amplitude of 1 nm. Approximately 4000 CSM indentations with a 3 μm spacing and a 100 nm maximum depth were generated over the entire region, forming a spatial property mapping with high resolution.

## 3. Results and Discussion

### 3.1. Initial Microstructure

**Figure 2:**
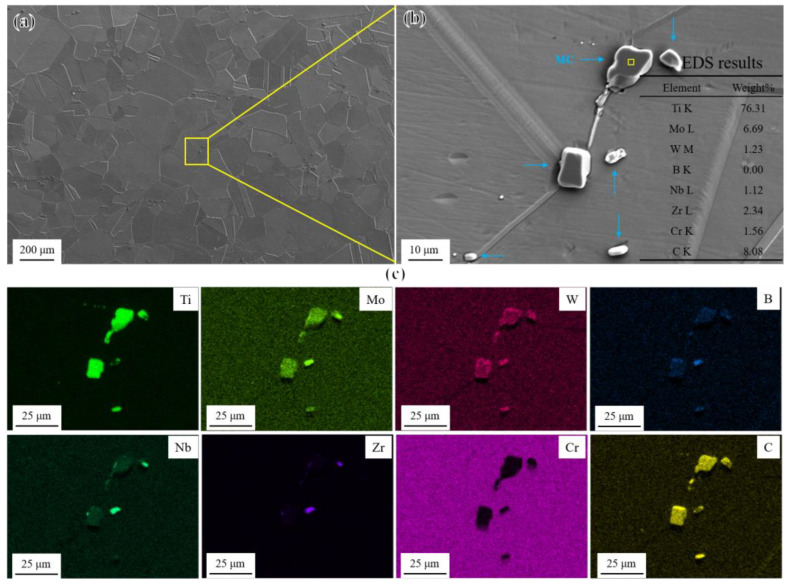
Microstructural characteristics of the novel alloy in the ST state: (**a**) overall microstructure; (**b**) morphology and EDS elemental analysis results of MC; and (**c**) EDS elemental mapping of MC in (**b**).

The overall microstructural characteristics of the novel alloy in the solution treatment (ST) state is shown in Figure 2a. The γ matrix was composed of equiaxed austenite grains with a grain size of approximately 150 μm (ASTM grade 3), containing numerous annealing twins and some randomly distributed blocky particles with a size of 1–10 μm, as shown in Figure 2b. According to the EDS elemental analysis results (Figure 2b) and elemental mapping (Figure 2c), the blocky particles were identified as MC carbides rich in Ti [30] and contained small amounts of carbide-forming elements such as Mo, W, and Nb. MC carbides with a melting point of about 1300 °C mainly formed during solidification and remained in the matrix because of incomplete dissolution and elimination during solution treatment [31]. In addition, the enrichment of Zr, B, and Nb was found in small-sized MC carbides, which may lead to HAZ liquation cracking in the novel alloy during subsequent welding [32,33].

**Figure 3:**
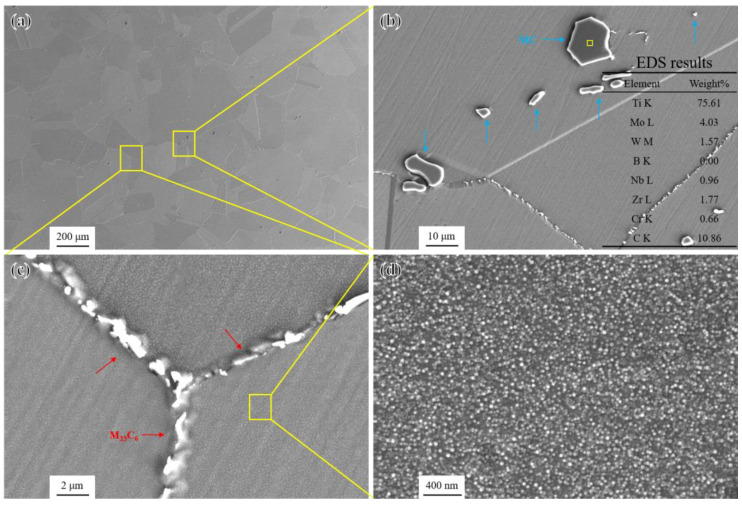
Microstructural characteristics in the SHT state: (**a**) overall microstructure; (**b**) morphology and EDS elemental analysis results of MC; (**c**) morphology of precipitates at grain boundaries; and (**d**) morphology of γ′ particles.

The overall microstructural characteristics of the novel alloy in the standard heat treatment (SHT) state are shown in Figure 3a. It can be seen that the grain size remained almost unchanged compared to the ST state. And the morphology and composition of blocky MC were also basically stable [34] (Figure 3b). Moreover, the microstructure in the SHT state included discrete small-sized precipitates distributed at grain boundaries (Figure 3c) and a large number of fine spherical γ′ particles with an average radius of about 12 nm uniformly distributed in grain interiors [30] (Figure 3d).

**Figure 4:**
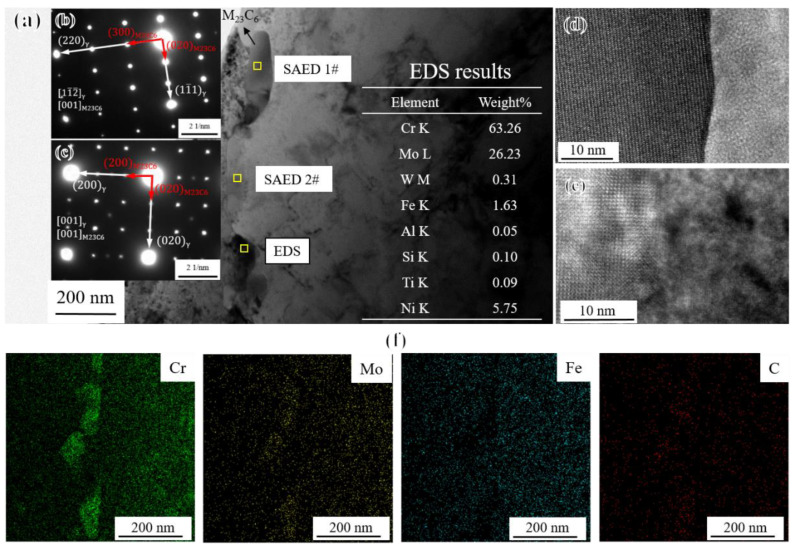
TEM analysis of M_23_C_6_: (**a**) bright field image at the grain boundary; SAED results of M_23_C_6_ and γ matrix of (**b**) position 1# and (**c**) position 2#; high-resolution image of (**d**) position 1# and (**e**) position 2#; and (**f**) EDS elemental mapping of M_23_C_6_ in (**a**).

According to the TEM analysis results and SAED patterns shown in Figure 4, the discrete precipitates with a size less than 1 um at grain boundaries were identified as M_23_C_6_ carbides rich in Cr and Mo [30], which maintained a coherent relationship with the γ matrix and precipitated along grain boundaries during the standard heat treatment. Generally speaking, discrete small-sized M_23_C_6_ carbides can hinder dislocation movement and grain boundary sliding, thus improving the creep rupture strength of the novel alloy to some extent [35].

**Figure 5:**
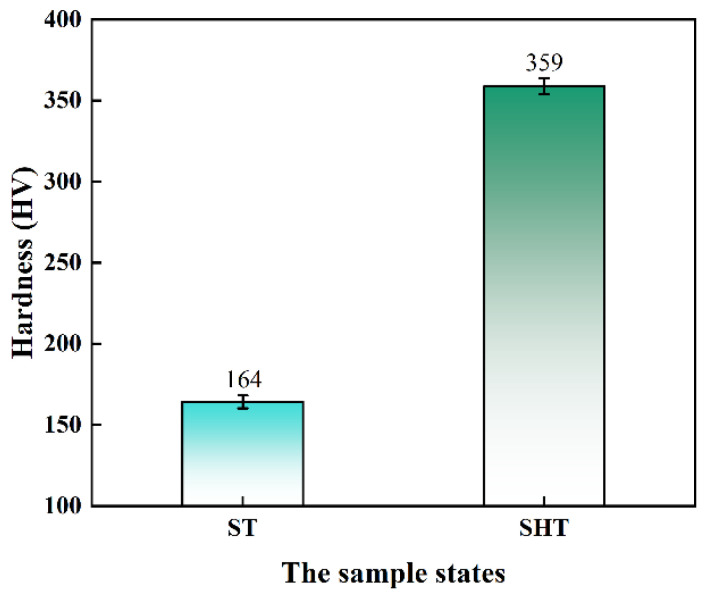
Comparison of hardness between ST and SHT states.

As a precipitation-strengthened superalloy, M_23_C_6_ carbides at grain boundaries have a certain influence on the strength of the novel alloy, but the fine spherical γ′ phase is the main strengthening phase [36]. The γ′ phase holds a coherent relation with the γ matrix [37] and enhances the strength of the novel alloy by hindering dislocation movement. Its size, content, shape and distribution have a decisive impact on the mechanical properties of the novel alloy [12,26]. As shown in Figure 5, the Vickers hardness of the novel alloy in the ST state was about 164 HV, while it reached about 359 HV in the SHT state, indicating that the strengthening effect of the fine γ′ particles precipitating during the standard heat treatment was extremely significant.

### 3.2. Precipitation Behavior during Short-Term Aging Treatment

#### 3.2.1. Precipitation Behavior at Grain Boundaries

**Figure 6:**
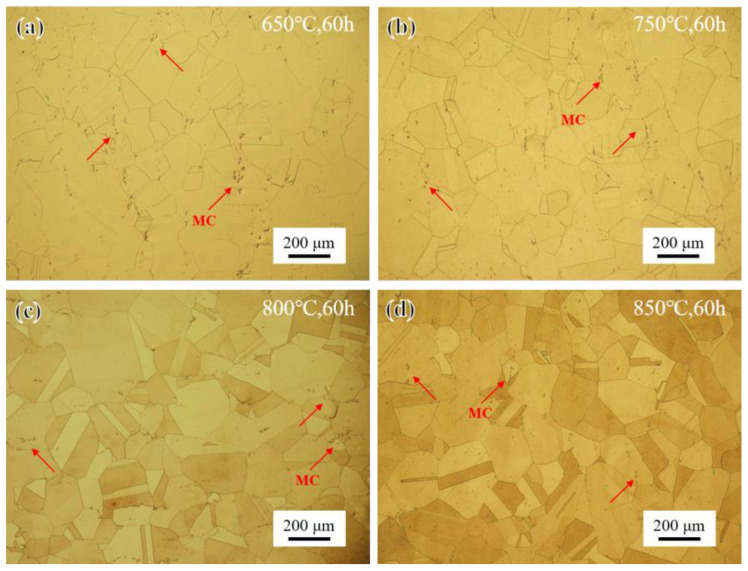
OM images of microstructure after short-term aging for 60 h: (**a**) 650 °C; (**b**) 750 °C; (**c**) 800 °C; and (**d**) 850 °C.

The microstructure of the novel alloy in the ST state after short-term aging treatment at different temperatures for 60 h is shown in Figure 6. Overall, the grain size remained at ASTM grade 3 at all four temperatures, consistent with the ST state. And there was no obvious change in the quantity and morphology of randomly distributed blocky MC carbides [31]. In addition, with the increase in the aging temperature, the contrast difference between different grains became larger and larger, with almost no difference at 650 °C, a slight difference at 750 °C, an obvious difference at 800 °C, and the most obvious difference at 850 °C, which is speculated to be caused by the precipitation of the γ′ phase.

**Figure 7:**
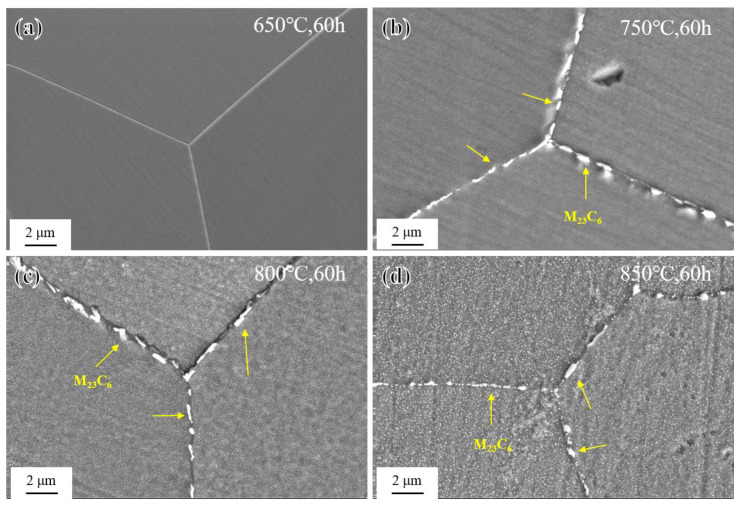
Morphology of M_23_C_6_ after short-term aging for 60 h: (**a**) 650 °C; (**b**) 750 °C; (**c**) 800 °C; and (**d**) 850 °C.

The morphology of M_23_C_6_ carbides at grain boundaries of the novel alloy after short-term aging for 60 h at different temperatures is shown in Figure 7. At 650 °C, there was almost no precipitation of M_23_C_6_ carbides, and the grain boundaries were very clean. At 750 °C, 800 °C, and 850 °C, as the aging time increased, M_23_C_6_ at grain boundaries gradually precipitated, aggregated, connected and grew, and finally formed a discrete chain-like distribution [21]. Furthermore, with the increase in the aging temperature, the precipitation behavior of the γ′ phase in grain interiors changed significantly, and the uniform distribution of fine γ′ particles could be gradually clearly seen, being completely invisible at 650 °C, blurred at 750 °C, relatively obvious at 800 °C, and most clearly visible at 850 °C, which was consistent with the contrast difference results of the OM images (Figure 6).

**Figure 8:**
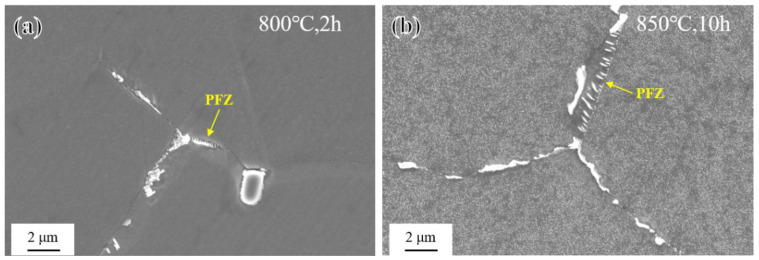
Abnormal coarsening behavior at grain boundaries during short-term aging: (**a**) 800 °C/2 h and (**b**) 850 °C/10 h.

It is particularly noteworthy that during short-term aging at 800 °C and 850 °C, as the aging time increased, the grain boundaries of the novel alloy exhibited abnormal coarsening behavior and formed precipitate-free zones (PFZs), as shown in Figure 8. Further related research will be elaborated in Section 3.4.

#### 3.2.2. Precipitation Behavior in Grain Interiors

**Figure 9:**
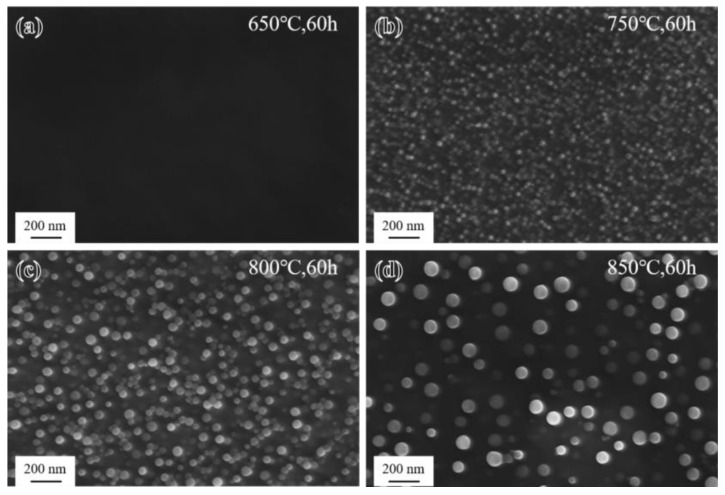
Morphology of the γ′ phase after short-term aging for 60 h: (**a**) 650 °C; (**b**) 750 °C; (**c**) 800 °C; and (**d**) 850 °C.

The morphology of the γ′ phase in the grain interiors of the novel alloy after short-term aging treatment for 60 h at different temperatures is shown in Figure 9. The size of the γ′ phase (the mean radii were invisible, at 11 nm, 18 nm, and 33 nm, respectively) increased rapidly with the aging temperature, while the volume fraction (invisible, 61%, 50%, and 30%, respectively) and the number density gradually decreased, which was attributed to the higher solubility of Ti and Al in the γ matrix at higher temperatures [38]. It is worth noting that the precipitation rate of the γ′ phase was very slow during short-term aging at 650 °C, so its size was extremely small and could not be observed. Therefore, only performing the first step of the aging treatment at 650 °C for 20 h cannot quickly achieve the effect of strengthening the novel alloy. In order to regulate the size and volume fraction of the γ′ phase to optimize the strength and toughness matching of the material, a second step of the aging treatment at 800 °C for 20 h was added to the novel alloy. When the novel alloy after the first step of aging was subjected to the second step of aging treatment (i.e., standard heat treatment), the γ′ phase could quickly and completely precipitate to reach equilibrium, and then gradually grow to reach a relatively suitable size and volume fraction (Figure 3d), thereby ensuring excellent mechanical properties of the material [26].

**Figure 10:**
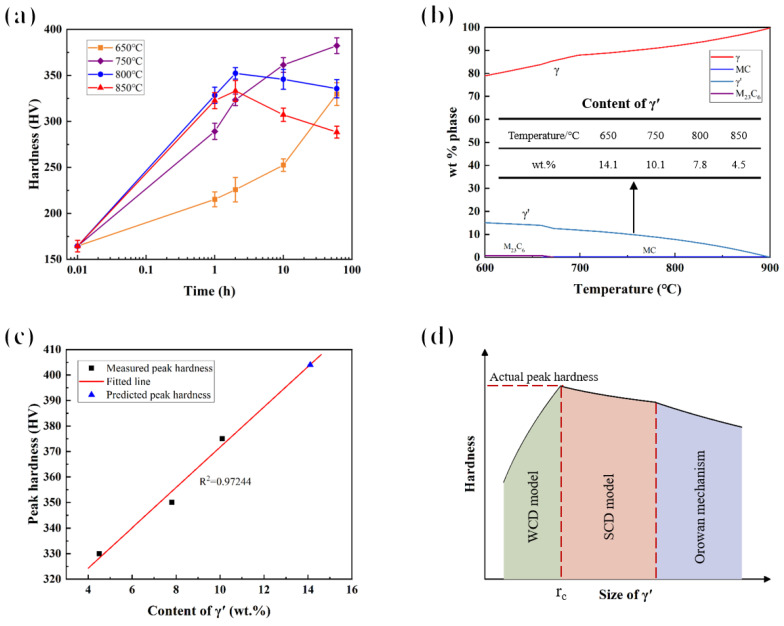
(**a**) A plot of hardness versus short-term aging time at different temperatures; (**b**) the equilibrium phase diagram of the main precipitates; (**c**) a plot of peak hardness versus the content of γ′; and (**d**) schematic diagram of hardness versus the size of the γ′ phase under different dislocation strengthening mechanisms.

The hardness of the specimens subjected to short-term aging at different temperatures for various time is shown in Figure 10a (the aging time of the specimen in the ST state is 0 h, which is set to 0.01 h for logarithmic plotting). At 650 °C, the hardness increased continuously with aging time; at 750 °C, the hardness continuously increased and gradually reached the peak hardness of 377 HV at 60 h; at 800 °C and 850 °C, the hardness first increased and then decreased, reaching the peak hardness (349 HV and 331 HV, respectively) at 2 h. To explain the differences in peak hardness at different temperatures, the equilibrium phase diagram of the main precipitates of the novel alloy was calculated using the thermodynamic calculation software JMatPro (version 7.0), as shown in Figure 10b. It can be seen that from 650 °C to 850 °C, as the temperature increases, the mass fraction of the γ′ phase gradually decreases, indicating a decrease in volume fraction. Therefore, the peak hardness of the novel alloy gradually decreased [39]. The relationship between the measured peak hardness (the measured peak hardness here is the maximum hardness in the measured value, not necessarily the actual peak hardness during aging) and the content of γ′ is fitted, as shown in Figure 10c. The fitting determination coefficient (R^2^) is 0.97244, indicating a good fitting effect, which once again proves that the variation in hardness was directly related to the precipitation behavior of the γ′ phase. Based on the fitted line, the peak hardness at 650 °C is predicted to be approximately 404 HV.

In γ′ precipitation-strengthened Ni-based superalloys, γ′ acts as a barrier to the movement of dislocations through a cutting mechanism and the Orowan bypassing mechanism [37] to achieve the strengthening effect. The type of cutting mechanism can be roughly subdivided into two groups, the weakly coupled dislocations (WCD) model [40] and the strongly coupled dislocations (SCD) model [41,42,43]. Combined with the precipitation behavior of γ′, the variation in hardness with time in Figure 10a can be explained through the dislocation strengthening mechanisms of γ′, as shown in Figure 10d. In the early stage of short-term aging, a large amount of γ′ was precipitated, and its strengthening effect was positively correlated with the volume fraction, so the hardness of the novel alloy increased rapidly. After a certain period of time, the precipitation of γ′ was almost over, and its volume fraction remained basically unchanged. At this time, the size of γ′ was still small, and the strengthening mechanism was the WCD model. The strengthening effect was enhanced with the increase in the size of γ′, and the hardness increased accordingly [44]. As the size of γ′ increased to reach the critical size *r_c_*, the strengthening mechanism began to transform into the SCD model. The strengthening effect of γ′ corresponding to the transition point was the strongest [45] and the hardness reached the actual peak hardness. With the further increase of the size of γ′, the hindering effect on dislocations gradually decreased and the hardness kept decreasing. When the critical resolved shear stress (CRSS) required for dislocations to bypass γ′ was smaller than the shear stress of the SCD model, the strengthening mechanism shifted to the Orowan mechanism, the strengthening effect became weaker [39], and the hardness also decreased accordingly.

**Figure 11:**
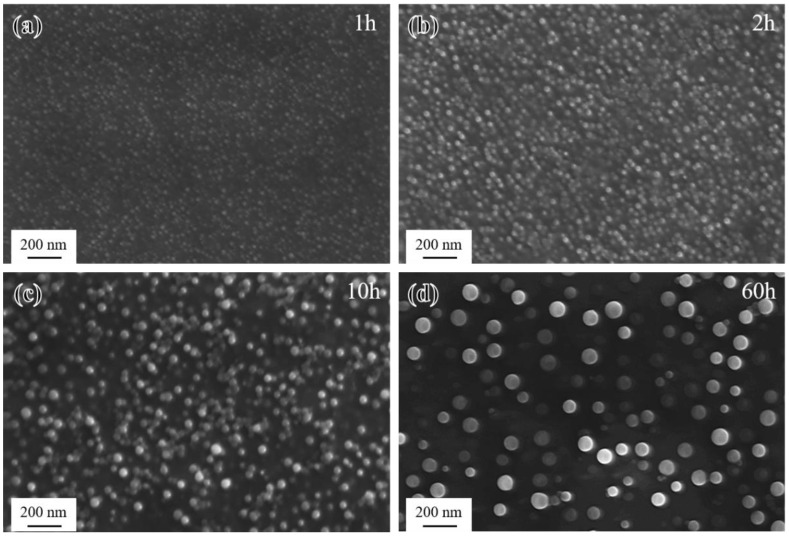
Morphology of γ′ after short-term aging at 850 °C: (**a**) 1 h; (**b**) 2 h; (**c**) 10 h; and (**d**) 60 h.

Conversely, the critical size of γ′ of the novel alloy can also be inferred from the change in hardness. The morphology of γ′ after short-term aging at 850 °C for 1–60 h is shown in Figure 11. The mean radii were 8 nm, 10 nm, 16 nm, and 33 nm, respectively. At 1 h, according to Figure 10a, the hardness was in the rising stage, indicating that the strengthening mechanism was the WCD model. Therefore, it can be judged that the critical size was larger than the average radius (8 nm) of γ′ at this time. When the aging time was 10 h, the hardness was in the decreasing stage, suggesting that the strengthening mechanism was the SCD model or the Orowan mechanism, so the critical size was smaller than the average radius (16 nm) of γ′ at this time. Consequently, it can be concluded that the critical size of achieving actual peak hardness during aging was within the range of 8–16 nm.

### 3.3. Precipitation Behavior during Long-Term Aging Treatment

#### 3.3.1. Precipitation Behavior at Grain Boundaries

**Figure 12:**
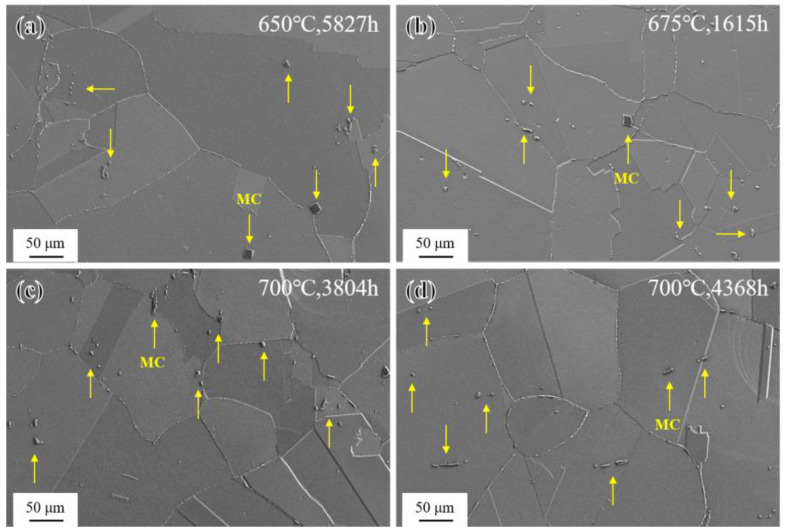
SEM images of microstructure after long-term aging: (**a**) 650 °C/5827 h; (**b**) 675 °C/1615 h; (**c**) 700 °C/3804 h; and (**d**) 700 °C/4368 h.

The microstructure of the novel alloy in the SHT state after long-term aging treatment at different temperatures is shown in Figure 12. Overall, the grain size was relatively unchanged during long-term aging and remained at ASTM grade 3, consistent with the SHT state. In addition, there were no obvious changes in the morphology and distribution of MC carbides [46].

**Figure 13:**
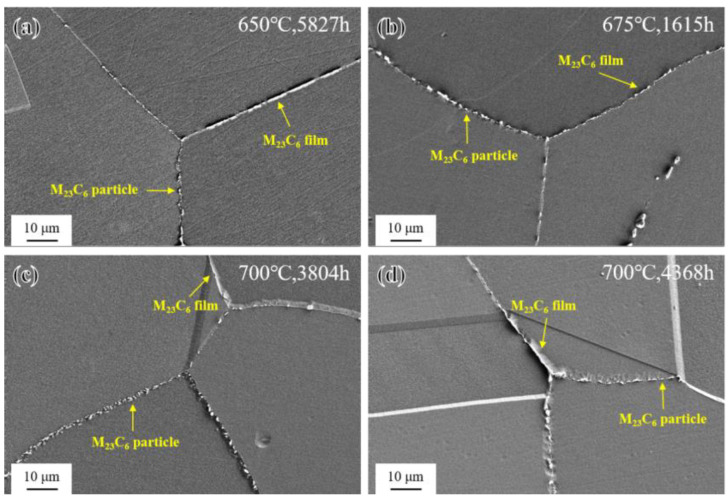
Morphology of M_23_C_6_ after long-term aging: (**a**) 650 °C/5827 h; (**b**) 675 °C/1615 h; (**c**) 700 °C/3804 h; and (**d**) 700 °C/4368 h.

The morphology of M_23_C_6_ at grain boundaries of the novel alloy after long-term aging at different temperatures is shown in Figure 13. Compared with the SHT state (Figure 3c), some discrete M_23_C_6_ had developed into a short and thin continuous film (about 10 μm long) at grain boundaries after long-term aging [13], which might reduce the impact toughness and creep rupture life of the novel alloy to some extent [18,47] due to reducing the pinning effect and facilitating crack nucleation and propagation at grain boundaries.

**Figure 14:**
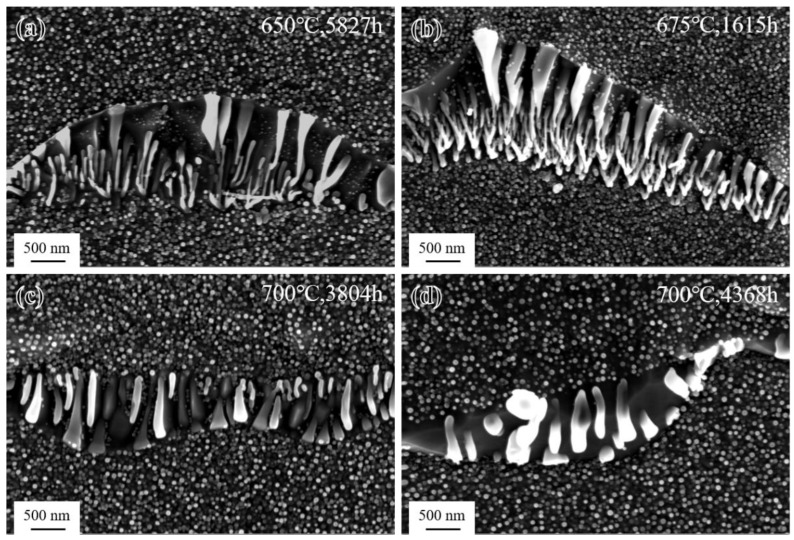
Abnormal coarsening behavior at grain boundaries during long-term aging: (**a**) 650 °C/5827 h; (**b**) 675 °C/1615 h; (**c**) 700 °C/3804 h; and (**d**) 700 °C/4368 h.

As shown in Figure 14, after long-term aging at 650–700 °C, the novel alloy also exhibited abnormal coarsening behavior at grain boundaries and formed PFZs, but it was different from that of short-term aging only occurring at 800 °C and 850 °C. Further related research will be elaborated in Section 3.4.

#### 3.3.2. Precipitation Behavior in Grain Interiors

**Figure 15:**
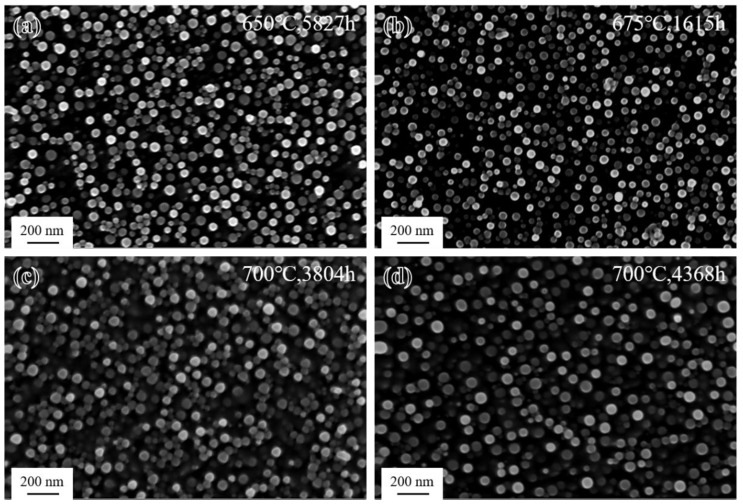
Morphology of γ′ phase after long-term aging: (**a**) 650 °C/5827 h; (**b**) 675 °C/1615 h; (**c**) 700 °C/3804 h; and (**d**) 700 °C/4368 h.

The morphology of the γ′ phase in the grain interiors of the novel alloy after long-term aging treatment at different temperatures is shown in Figure 15. It can be seen that γ′ particles (the mean radii were 21 nm, 18 nm, 29 nm, and 30 nm, respectively) maintained a spherical shape and uniform distribution during long-term aging but underwent a certain degree of coarsening process at all three temperatures compared to the SHT state (Figure 3d). Comparing Figure 15a,b, it can be seen that the average size of γ′ particles at 650 °C was larger, which was related to the much longer aging time at this temperature. Figure 15c,d show that the size of γ′ particles gradually increased as the aging time increased during long-term aging at 700 °C, while the number density gradually decreased [13]. The aging time at 700 °C was shorter than that at 650 °C, but the temperature was significantly higher, so the size of γ′ particles was relatively larger.

**Figure 16:**
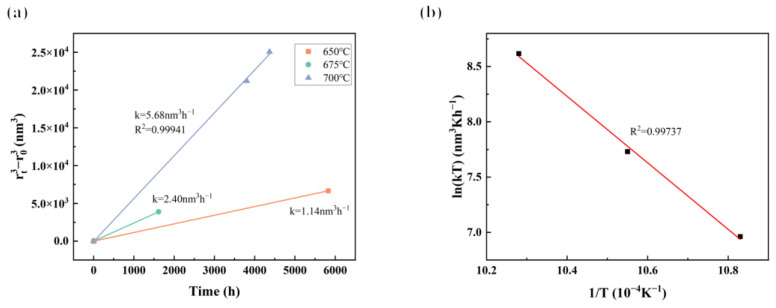
(**a**) A plot of the size of the γ′ phase versus long-term aging time at different temperatures and (**b**) the relationship between temperature (*T*) and the coarsening rate (*k*).

Many findings [13,48,49,50,51,52] have confirmed that the coarsening behavior of γ′ particles in Ni-based superalloys follows the diffusion-controlled [53] Lifshitz–Slyozov–Wagner (LSW) growth kinetics model. The LSW model states that when the chemical composition and volume fraction of γ′ particles remain constant at the same temperature, the size of γ′ particles varies according to the formula $r_{t}^{3}-r_{0}^{3}=kt$ [54,55], where *r_t_* and *r*_0_ are the average radius of γ′ particles at aging times *t* and 0, respectively, and *k* is the coarsening rate [49]. The cubic of the average size of γ′ particles after various long-term aging times at three temperatures is plotted as a function of time in Figure 16a. According to the result at 700 °C, it can be seen that the cubic of the average radius of γ′ particles increases linearly with the aging time and R^2^ of the fitted line is 0.99941, which indicates that the coarsening behavior of γ′ particles in the novel alloy during long-term aging also followed the diffusion-controlled LSW model.

During long-term aging, it was mainly the reduction in the specific free energy at the γ′/γ interface [49] that drove the coarsening of γ′ particles. As the aging progressed, due to the gradual increase in lattice misfit, the coherency of the γ′/γ interface was disrupted and the interfacial energy increased [56], resulting in an enhanced driving force for the coarsening of γ′ particles. The coarsening behavior of γ′ particles was then promoted to occur at the expense of interfacial energy reduction. Actually, although immobile in the γ matrix, γ′ particles tended to reduce the interfacial energy through the diffusion of the forming elements Al and Ti from smaller particles to larger particles, which meant promoting the coarsening of large particles by dissolving small γ′ particles [31]. Therefore, the number density of γ′ particles decreased but the average size increased. From the slope *k* of the line fitted by the LSW model, it can be seen that the higher the temperature, the faster the coarsening of γ′ particles.

In addition, after obtaining the coarsening rate *k* at the three temperatures according to Figure 16a, the coarsening activation energy *Q* of γ′ particles can also be calculated by the formula *ln*(*kT*) = constant −Q/*RT* [57], where *T* is the absolute temperature and *R* is the ideal gas constant of 8.314 J/(mol K). The relationship between *ln*(*kT*) and *1*/*T* is shown in Figure 16b. The slope of the fitted line (R^2^ = 0.99737) is −3.01, so the coarsening activation energy of γ′ particles is calculated to be 250.3 kJ/mol, which is close to the volume diffusion activation energies of both Ti and Al in Ni (257 and 270 kJ/mol, respectively) [58]. Similar activation energy values for γ′ coarsening have also been reported in some Ni-based superalloys [48,49,58,59]. This result again suggests that the coarsening of γ′ particles was mainly achieved by the volume diffusion of Al and Ti, and the coarsening activation energy was dominated by the mole fractions of Ti and Al atoms in the γ matrix [12].

**Figure 17:**
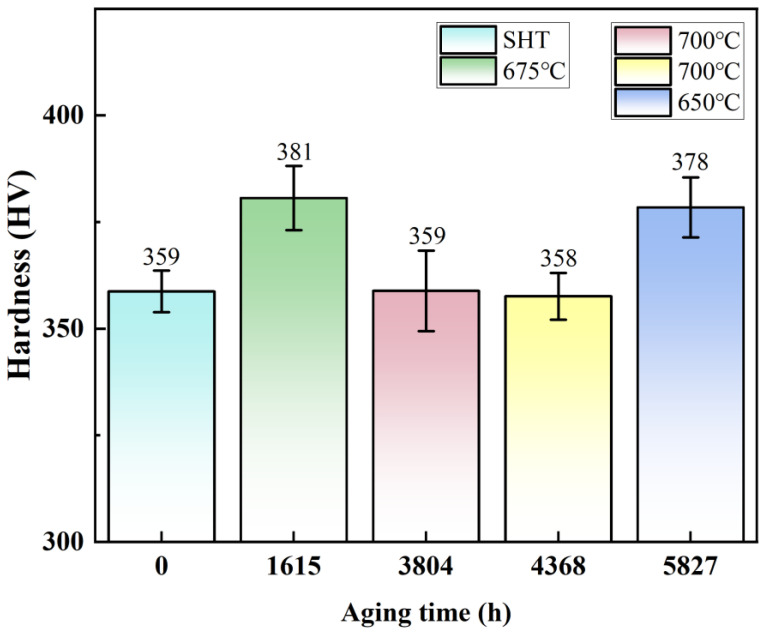
Comparison of hardness before and after long-term aging at different temperatures.

The hardness measurement results of the novel alloy after long-term aging at different temperatures are shown in Figure 17. According to the results of short-term aging, the critical size of the novel alloy was within the range of 8–16 nm. As shown in Figure 15, the average radius of γ′ particles after long-term aging at 675 °C for 1615 h was the smallest at 18 nm, exceeding the critical size. Therefore, its hardness (381 HV) was in the decreasing stage shown in Figure 10d, and the strengthening mechanism was the SCD model or the Orowan mechanism. Similarly, after long-time aging at 650 °C for 5827 h and 700 °C for 3804 h and 4368 h, the average radii of γ′ particles also exceeded the critical size, the hardness was in the decreasing stage, and the larger the average size of γ′ particles, the lower the hardness. In addition, it can also be judged that the hardness of the SHT state (359 HV) was in the rising stage, and its strengthening mechanism was the WCD model, so the average radius of γ′ particles (12 nm) had not reached the critical size. Consequently, it can be further concluded that the critical size of achieving actual peak hardness was within the range of 12–16 nm.

### 3.4. Abnormal Coarsening Behavior at Grain Boundaries

#### 3.4.1. Characterization of PFZs

**Figure 18:**
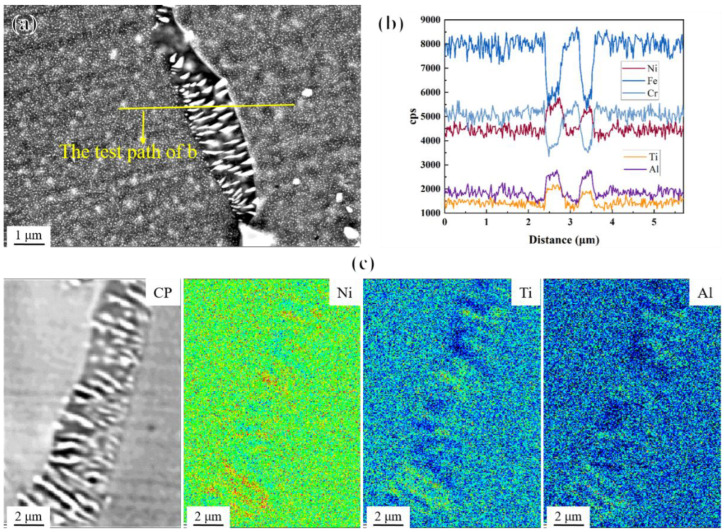
(**a**) SEM image of PFZ; (**b**) EDS elemental analysis along the test path in (**a**); and (**c**) EPMA elemental mapping of PFZ.

In the study of precipitates’ evolution during the high-temperature aging treatment of the novel alloy mentioned above, abnormal coarsening behavior at grain boundaries occurred during short-term aging at 800 °C and 850 °C (Figure 8), and during long-term aging at 650 °C, 675 °C, and 700 °C (Figure 14). Partial grain boundaries migrated, and precipitate-free zones (PFZs) [24,25,31,60,61,62,63] were formed. Actually, PFZs used to specifically describe the coarsened grain boundary features were not truly free of second phases. On the contrary, while a large number of previously existing fine γ′ phases disappeared, some coarsened and elongated rod-like particles were formed in these regions, so PFZs are also known as precipitate-denuded regions or coarsened zones [64]. From the EDS elemental analysis results (Figure 18b) and EPMA elemental mapping (Figure 18c), it can be seen that the rod-like particles were rich in Ni, Al, and Ti elements, suggesting that they were likely abnormally coarsened γ′ precipitates.

**Figure 19:**
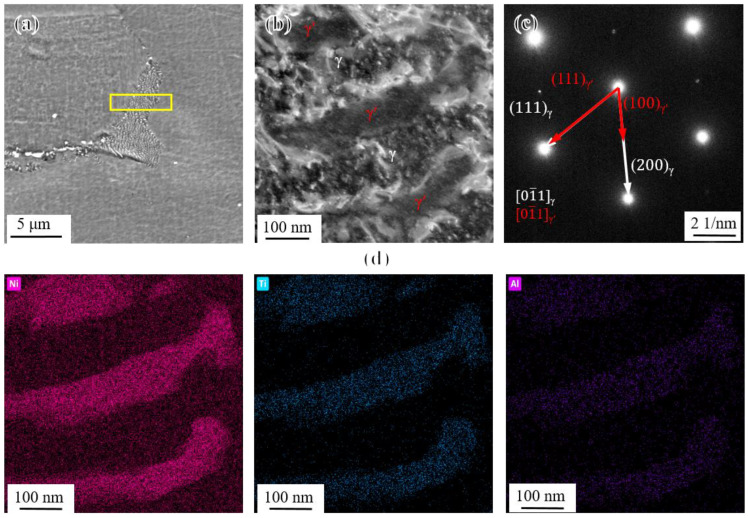
(**a**) Microstructure of PFZ taking the position of the TEM sample prepared by FIB; (**b**) bright field image of the TEM sample; (**c**) SAED patterns of rod-like γ′ phase and γ matrix; and (**d**) EDS elemental mapping of rod-like γ′ phase.

To confirm the speculation about the rod-like particles, the region containing the PFZ (as indicated by the yellow frame) in Figure 19a was cut and sampled using the FIB technique for TEM observation, and the bright field image was obtained, as shown in Figure 19b. The SAED patterns shown in Figure 19c indicate that the rod-like phase held a coherent relation with the γ matrix and involved the same face-centered cubic (FCC) crystal orientation. Combined with the EDS elemental mapping of Ni, Ti, and Al shown in Figure 19d, it can be confirmed that the rod-like phase was indeed an abnormally coarsened γ′ phase [62,63]. In addition, there was no presence of the η phase in PFZs, which could be attributed to the low ratio of Ti to Al (Ti: Al = 1.43) in the novel alloy [13].

#### 3.4.2. Formation of PFZs

**Figure 20:**
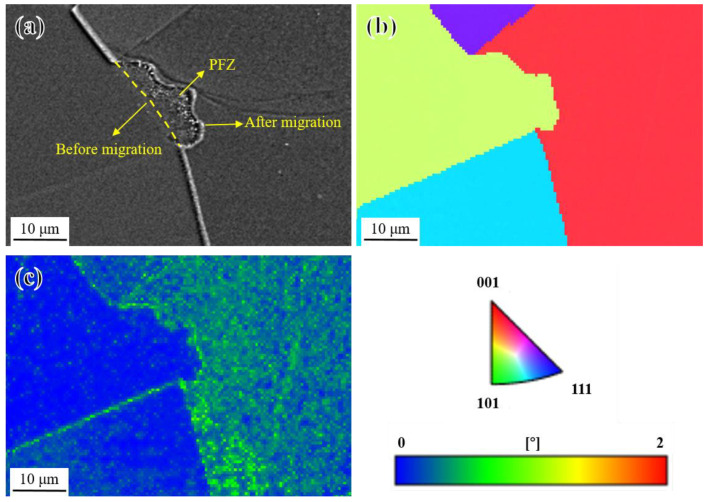
EBSD analysis of PFZ; (**a**) SEM image; (**b**) IPF map of (**a**); and (**c**) KAM map of (**a**).

In order to clarify the formation of the PFZ, the crystal orientation and dislocation density distribution of grains on both sides of the coarsened grain boundary were investigated by EBSD. As shown in Figure 20a,b, the crystal orientation of the PFZ remained consistent with the left grain, indicating that the grain boundary migrated from the left grain to the right grain. Furthermore, as shown in Figure 20c, the kernel average misorientation (KAM) of the left grain was obviously lower than that of the right grain. Generally speaking, the larger the KAM, the higher the geometrically necessary dislocation (GND) density [65] and the higher the strain gradient and stored strain energy inside the crystal [66]. Therefore, it can be inferred that grain boundaries migrated from grains with a lower dislocation density to grains with a higher dislocation density, forming PFZs to reduce the strain energy near the grain boundaries [60,67].

In fact, similar abnormal coarsening behavior at grain boundaries has also been observed in many Ni-based superalloys, including TG700A [31,60], 617B [61], 740H [62], Nimonic 263 [63], etc. And PFZs were also formed, with the rod-like γ′ phase appearing inside. However, most researchers have discovered this phenomenon during high-temperature creep (especially in the third stage of creep), and believe that PFZs only occur when both high-stress or strain and high-temperature conditions are simultaneously present and when the stress or strain is applied for a sufficiently long period of time. Accordingly, several mechanisms including Nabarro–Herring creep, grain boundary carbide formation, and grain boundary sliding or migration have been proposed to explain the formation of PFZs. Comparatively, the occurrence during aging is rarely mentioned. The lack of external stress resulted in the previous mechanisms no longer being fully applicable to the abnormal coarsening behavior at grain boundaries discovered during aging.

**Figure 21:**
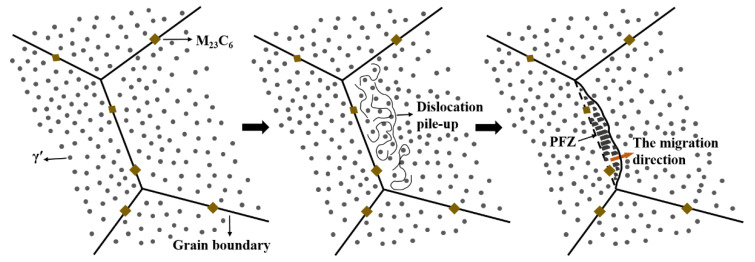
Schematic diagram of the formation of PFZ.

Based on the above research results, this paper proposes a new mechanism of grain boundary migration and the discontinuous coarsening reaction to explain the abnormal coarsening behavior at grain boundaries during aging of the novel alloy, as shown in Figure 21. During the forging and heat treatment processes of large thick-walled structural components, high residual stress or strain is easily generated inside the material due to uneven temperature distribution and plastic deformation [68,69]. During aging, the significant decrease in the lattice constant [12] results in strong negative creep behavior, causing high shrinkage stress within the novel alloy. Under the coupling effect of high temperature and high stress or strain, dislocation pile-up occurs due to a significant difference in orientation between adjacent grains, and the grain boundary migrates towards the grain with a higher dislocation density [70]. After grain boundary migration occurs, the rapid diffusion and local segregation of Al and Ti along the moving grain boundaries lower the solidus temperature of the γ′ phase, causing the fine spherical γ′ particles in the area through which the grain boundary migration passes to dissolve [31]. At the same time, the local solute atoms are in a supersaturated state. Due to the stability of the composition, the new γ′ phase directionally precipitates along the grain boundary migration direction and discontinuous coarsening reactions occur under the Oswald-like mechanism, forming rod-like abnormally coarsened and elongated γ′ precipitates inside the area passed by the migrating grain boundary [71,72]. Finally, PFZs lacking fine spherical γ′ but containing the coarse rod-like γ′ phase are formed, manifested as abnormally coarsened grain boundaries through metallographic observation.

The mechanism proposed above can explain why abnormal coarsening behavior at grain boundaries occurred under different conditions for short-term aging and long-term aging. When the aging temperature reached 800 °C or above, the speed of grain boundary migration and element diffusion was greatly improved, so abnormal coarsening behavior at grain boundaries could occur in just a few hours, as shown in Figure 8. When the temperature dropped to 650 °C, due to the cumulative effect, the behavior could also occur when the aging time reached several thousand hours, as shown in Figure 14.

Overall, there are still some limitations in the research on the mechanism. For instance, residual stress and negative creep shrinkage stress are only qualitatively described and not quantitatively measured. The study on the formation conditions of PFZs is still insufficient, and subsequent creep experiments can be considered to quantitatively study the formation conditions and evolution of PFZs. In addition, most of the key components of the A-USC units need to be welded, and considering that the welding residual stress, welding metallurgical behavior, and post-weld heat treatment (PWHT) may affect the formation of PFZs, it is also necessary to study the weldability of the novel alloy.

#### 3.4.3. Influence of PFZs

**Figure 22:**
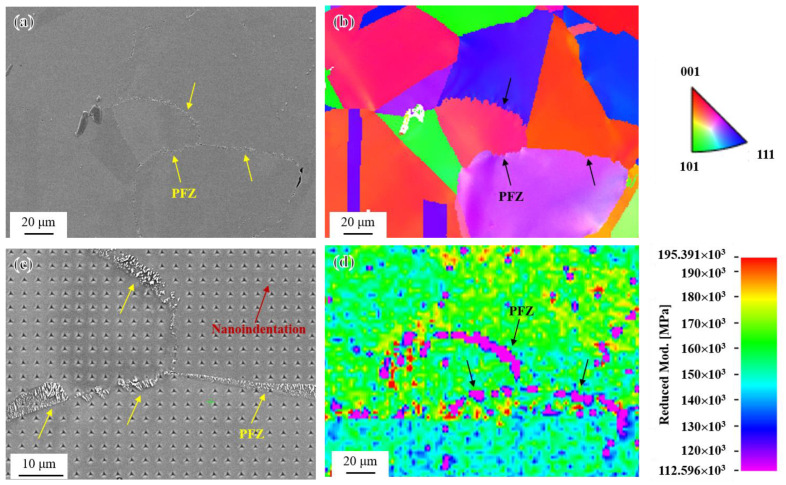
In situ nanomechanical test of PFZs: (**a**) SEM image; (**b**) IPF map of (**a**); (**c**) local nanoindentation image of (**a**); and (**d**) reduced modulus map of (**a**).

After the occurrence of abnormal coarsening behavior at grain boundaries in the novel alloy, PFZs in the coarsened grain boundaries lacked a sufficient amount of uniformly distributed spherical γ′ particles for strengthening, and the coarsened and elongated rod-like γ′ phase may even have had a weakening effect. Therefore, it is speculated that the mechanical properties of PFZs were weaker compared to the intragranular regions. A high-resolution nanoindentation test in a 220 μm × 160 μm region was conducted to compare the mechanical properties of PFZs and near grains. The SEM image and inverse pole figure (IPF) of the selected area are shown in Figure 22a,b, respectively. Figure 22c is a local nanoindentation image of the area after the completion of the test. It can be seen from Figure 22d corresponding to Figure 22a that the reduced modulus in PFZs was significantly lower than that in the intragranular regions on both sides, which indicated that PFZs of the novel alloy indeed had poor mechanical properties and might fail first during service [24,25,31,62].

**Figure 23:**
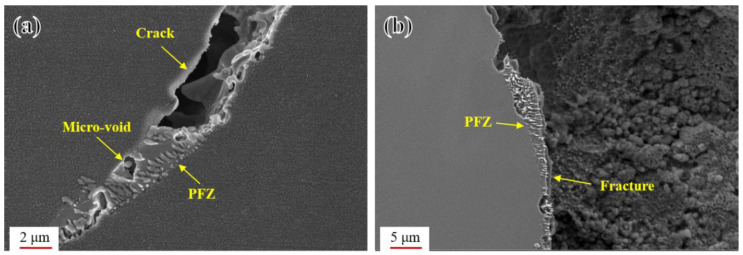
Cross-section SEM images of the creep rupture specimen at 700 °C/230 MPa: (**a**) the relationship between the PFZ and the crack. and (**b**) the relationship between the PFZ and the fracture.

Observation of the creep rupture specimen at 700 °C/230 MPa confirms that abnormal coarsening behavior at grain boundaries had a significant impact on the creep rupture performance of the novel alloy. From Figure 23a, it can be seen that creep micro-voids originated from the PFZ [31], gradually grew and expanded, connected to form cracks, and finally promoted the rupture, as shown in Figure 23b. Consequently, combined with the analysis of the weaker properties of PFZs mentioned above, it can be inferred that the abnormal coarsening behavior at grain boundaries was an important reason for the earlier failure of the novel alloy during the high-temperature creep rupture test. Inhibiting this behavior will greatly improve the creep rupture performance.

## 4. Conclusions

In this paper, the precipitation behavior of a novel Ni-Fe-based superalloy in the solution treatment (ST) state and the standard heat treatment (SHT) state during aging treatment was studied through microstructure characterization and mechanical property testing, and a detailed analysis of abnormal coarsening behavior at gain boundaries was provided. The following conclusions are made based on the research results above:

(1) When the novel alloy was in the ST state, the grain size was at ASTM grade 3, and blocky MC carbides rich in Ti were randomly distributed in grain interiors and at grain boundaries. When the novel alloy was in the SHT state, the grain size remained almost unchanged, and MC was also stable. Discrete M_23_C_6_ carbides rich in Cr precipitated at grain boundaries, and numerous uniformly distributed fine spherical γ′ particles precipitated in grain interiors, which directly led to a significant increase in hardness.
(2) After short-term aging of the novel alloy in the ST state, there was no obvious change in the grain size and MC. At 650 °C, almost no precipitates were observed at grain boundaries, and the γ′ phase precipitated slowly, resulting in a small quantity and size. At 750 °C, 800 °C, and 800 °C, discrete M_23_C_6_ and the fine γ′ phase rapidly precipitated and gradually grew. After the complete precipitation of the γ′ phase, the hardness reached the peak hardness when the average radius of the γ′ phase increased to the critical size, and then gradually decreased with the increase in the size of the γ′ phase.
(3) After long-term aging of the novel alloy in the SHT state, the grain size and MC remained almost unchanged. Discrete M_23_C_6_ at grain boundaries underwent a transition to continuous films. The size of the γ′ phase in grain interiors significantly increased, and the coarsening behavior followed the Lifshitz–Slyozov–Wagner (LSW) model with a coarsening activation energy of 250.3 kJ/mol. The hardness increased at 650 °C and 675 °C and slightly decreased at 700 °C, while all were below the peak value, which was related to the fact that the average radii of γ′ exceeded the critical size (12–16 nm).
(4) The novel alloy exhibited abnormal coarsening behavior at grain boundaries during both short-term and long-term aging. Partial grain boundaries migrated, and precipitate-free zones (PFZs) were formed. It was confirmed that the grain boundary migrated from the grain with a lower KAM to the grain with a higher KAM, forming abnormally coarsened and elongated rod-like γ′ particles along the migration direction. It is proposed that the formation mechanism of PFZs is strain-induced grain boundary migration and a discontinuous coarsening reaction.
(5) PFZs had weaker mechanical properties compared to nearby grains and were considered to be potential crack sources during the creep rupture test, leading to an earlier failure of the novel alloy. To ensure the service performance of the novel alloy, it is necessary to inhibit the abnormal coarsening behavior at grain boundaries. Based on the study results of the formation mechanism of PFZs, relevant measures should consider optimizing grain boundary character distribution (GBCD), improving composition segregation, and reducing residual stress through appropriate heat treatment. Once the abnormal coarsening behavior is controlled, the performance will be greatly improved, and it is predicted that the much cheaper novel alloy may be able to match the durability of Ni-based superalloys, such as 617B and 740H, and further promote the development of practical applications for thermal power units above 650 °C.

## Figures and Tables

**Table 1:** Chemical composition of the novel alloy.

Elements	Fe	Ni	Cr	Al + Ti	Mo	C + W + Co + B + Nb + Zr
wt.%	42.0	Bal.	16.0	3.4	0.5	0.7

## Data Availability

The raw data supporting the conclusions of this article will be made available by the authors upon request.
